# The biomedical piglet: establishing reference intervals for haematology and clinical chemistry parameters of two age groups with and without iron supplementation

**DOI:** 10.1186/s12917-017-0946-2

**Published:** 2017-01-17

**Authors:** Domenico Ventrella, Francesco Dondi, Francesca Barone, Federica Serafini, Alberto Elmi, Massimo Giunti, Noemi Romagnoli, Monica Forni, Maria L. Bacci

**Affiliations:** Department of Veterinary Medical Sciences, Alma Mater Studiorum, University of Bologna, Via Tolara di Sopra 50, 40064 Ozzano dell’Emilia, BO Italy

**Keywords:** ADVIA 2120, Clinical chemistry, Haematology, Reference intervals, Swine

## Abstract

**Background:**

The similarities between swine and humans in physiological and genomic patterns, and the great correlation in size and anatomy, make pigs extremely useful in preclinical studies. New-born piglets can represent a model for congenital and genetic diseases in new-born children. It is known that piglets may have significant differences in clinicopathological results compared to adult pigs. Therefore, adult laboratory reference intervals cannot be applied to piglets. The aim of this study was to compare haematological and chemical variables in piglets of two ages and determinate age-related reference intervals for commercial hybrid young pigs.

Blood samples were collected under general anaesthesia from 130 animals divided into five- (P5) and 30- (P30) day-old piglets. Only P30 animals were treated with parenteral iron after birth. Samples were analysed using automated haematology (ADVIA 2120) and chemistry analysers, and age-related reference intervals were calculated.

**Results:**

Significant higher values of RBC, Hb and HCT were observed in P30 animals when compared to P5, with an opposite trend for MCV. These results were associated with a reduction of the RBC regeneration process and the thrombopoietic response. The TSAT and TIBC were significantly higher in P30 compared to P5; however, piglets remained iron deficient compared to adult reference intervals reported previously.

**Conclusions:**

In conclusion, this paper emphasises the high variability occurring in clinicopathological variables between new-born and 30-day-old pigs, and between piglets and adult pigs. This study provides valuable reference data for piglets at precise ages and could be used in the future as historical control improving the Reduction in animal experiments, as suggested by the 3Rs principle.

**Electronic supplementary material:**

The online version of this article (doi:10.1186/s12917-017-0946-2) contains supplementary material, which is available to authorized users.

## Background

The interest in the pig as an animal model for experimental medicine can be traced back to Galen, in 1586 [1]. One reason for this interest is the strong similarities between the pig and the human in both physiological [2] and genomic [3] patterns. In addition, size and anatomy can be easily related to the development stages of people, making the pig the perfect preclinical model for human diseases [4, 5], surgical techniques and, more recently, for transplantation research [6–8]. When compared to other models such as mice or rats, the pig has a longer lifespan of 10–15 years [9], so disease progression is more similar to that seen in humans [1]. Furthermore, in the neonatal period, pigs represent an accurate model for studying congenital and genetic diseases in humans [10]. Piglets can even represent a good model for the preterm neonate, as they show similar anthropometric and physiological characteristics [11].

However, age differences, even within the same species, significantly affect the comparison of some developmental patterns, especially in extremely young subjects. Therefore, these processes need to be thoroughly investigated in order to create an accurate and standardised preclinical model and to help reduce and refine experimental protocols. As an important example, iron deficiency, which is one of the most common nutritional defects during the neonatal period in mammals [12, 13], is extremely common in swine, due to the high reproductive performance required of these animals. Unless given iron supplements, piglets may develop iron-deficiency a few days after birth [14, 15]. This condition occurs regardless of the breed and management system, and is the result of interactions of several factors including low levels of iron stores, increased requirements, poor exogenous supply and immaturity of absorption mechanisms [15, 16]. Similarly, iron requirements cannot be completely fulfilled by hepatic reserves and milk consumption due to the constant request for larger litter sizes, higher birth weights and faster growth that result in a greater blood volume and red blood cell (RBC) count [17]. It is therefore mandatory to supplement piglets with exogenous iron to prevent dangerous deficiency [18]. This procedure may interfere with several clinical chemistry parameters [19] and is the reason why it is very inaccurate to evaluate piglets based on the clinicopathological findings of older pigs. Therefore, it is extremely important to have specific age-related reference intervals for both haematological and chemical variables for piglets. Some values have been described in a single litter of Duroc x Jersey piglets [20], but the small number of animals and the lack of information about iron supplementation make them hard to rely on.

The aim of this study was to evaluate haematological and chemical variables in two groups of healthy hybrid piglets of different ages. Secondary objectives were to establish age-related reference intervals (RI) for both haematology and clinical chemistry variables and to evaluate the iron profile in new-born piglets without exogenous supplementation (5 days old) and young pigs administered with exogenous iron within 3 days after birth (30 days old).

## Methods

### Animals

All of the animals were Italian Large White x Duroc x Landrace commercial hybrids used in our facility. We only selected control and/or pre-treated animals previously enrolled in other experimental protocols and approved by the local ethical committee to be part of this study. The above mentioned protocols included blood tests to evaluate the animals, and we decided to work on the obtained data set of blood values.

We analysed piglets at two different time points: P5 were five-day-old piglets born in our facility that had not received any iron supplementation before blood sampling and were not neutered; P30 were 30-day-old pigs that were transferred to our piggery on the day of weaning (28th day of life) and were administered a single iron injection (100 mg IM; Endofer, FATRO, Italy) within the first 72 h after birth, and males were neutered. None of the animals was included in both age groups. In order to rule out any possible variation in genetic line and management, both pregnant sows and pigs were born and raised in the same farm. All of the animals were housed in multiple stalls and fed with a standard swine diet; P5 were housed in the farrowing crate with the sow until weaning. Body weight (kg) was measured in P5 and P30 and recorded.

A total of one hundred-thirty animals were included in the study; 74/130 (57%) were females, while 56/130 (43%) were males. Body weight was 2.3 (1.2–3.8) kg in P5 and 8.0 (5.3–11.2) kg in P30. For haematologicalal analyses, samples from 130 animals were available: 66 P5 and 64 P30. For chemistry evaluations, samples from 119 animals were available: 56 P5 and 63 P30.

### Blood sample collection and analyses

Blood samplings were performed on day 5 (P5) or 30 (P30) under general anaesthesia, using an advanced anaesthesia delivery unit (Datex-Ohmeda ADU S/5, GE Healthcare, USA), achieved by inhalation induction with sevoflurane (Sevoflo, Abbott Laboratories, Chicago, USA). No premedication was performed in order to avoid blood alterations due to injected drug adsorption. After oro-tracheal intubation, anaesthesia was maintained with 4 ± 0.5% sevoflurane in a 1:1 oxygen/air mixture. Samples were obtained from the femoral artery using a 21 G butterfly needle and a vacuum system; tubes with K_3_EDTA anticoagulant, citrate and clot activator were used. The total volume of withdrawn blood was approximately 10 ml, which was considered completely safe and negligible for these animals.

Blood samples (K_3_EDTA tubes) were analysed within 30 min from collection; serum (clot activator tubes) and citrate plasma (citrate tubes) were obtained by centrifugation (10 min at 3000 × g) within 1 h and analysed or stored at −80 °C until analysis.

Complete blood count (CBC) was performed with a new automated haematology analyser (ADVIA 2120, Siemens Healthcare Diagnostics, Tarrytown NY, USA) that combines classic haematological variables with individual cell indices. The variables evaluated in our study were haematocrit value (HCT), haemoglobin concentration (Hb), cellular haemoglobin content (CH), cellular haemoglobin content of mature red blood cells (CHm), red blood cells count (RBC), mean corpuscular volume (MCV), mean corpuscular volume of mature RBCs (MCVm), mean corpuscular haemoglobin concentration (MCHC), mean corpuscular haemoglobin (MCH), corpuscular haemoglobin concentration mean (CHCM), corpuscular haemoglobin concentration mean of mature RBCs (CHCMm), haemoglobin concentration distribution width (HDW), haemoglobin concentration distribution width of mature RBC (HDWm), RBC distribution width (RDW) and mature RBC distribution width (RDWm). Total white blood cell (WBC) count and differential WBC count were also performed. Platelet indices were analysed and included platelet count (PLT), mean platelet volume (MPV), PLT volume distribution width (PDW), plateletcrit (PCT), mean PLT component (MPC), platelet component distribution width (PCDW), mean PLT mass (MPM) and platelet mass distribution width (PMDW).

In addition to the above mentioned variables, we evaluated the following reticulocyte indices: absolute reticulocyte count (Retic), percentage of reticulocytes (%Retic), average size of reticulocytes (MCVr), average cell haemoglobin concentration (CHCMr), average haemoglobin content (CHr), distribution width of reticulocyte cell size (RDWr), distribution width of CHCMr (HDWr), percentage of microcytic reticulocytes (%Micro-r), percentage of macrocytic reticulocytes (%Macro-r), percentage of hypochromic reticulocytes (%Hypo-r), percentage of hyperchromic reticulocytes (%Hyper-r), percentage of reticulocytes with a low CH (%LowCHr), percentage of reticulocytes with a high CH (%HighCHr), CHr-CHm (CH delta), CHCMr-CHCMm (CHCM delta), CHDWr-CHDWm (CHDW delta), HDWr-HDWm (HDW delta), MCVr-MCVm (MCV delta), and RDWr-RDWm (RDW delta). The haematologicalal evaluation was completed by a blood smear microscopic examination using Romanovsky staining.

All chemistry analyses were carried out on an automated chemistry analyser (Olympus AU 400, Beckman Coulter/Olympus) and included aspartate transaminase (AST), alanine transaminase (ALT), alkaline phosphatase (ALP), creatinine, urea, glucose, total proteins (TP), albumin, albumin to globulin ratio (A/G), sodium, potassium, total iron (TI), unsaturated iron binding capacity (UIBC), total iron binding capacity (TIBC) and TIBC saturation (TSAT). Total iron and UIBC were measured using colorimetric methods (Iron Ferene, KAL 002, Olympus/Sentinel Diagnostics, Milan, Italy; UIBC OSR61205, Olympus/Beckman Coulter, O’Callaghan’s Mills, Ireland). Total iron binding capacity and TSAT were calculated as follows: TIBC = TI + UIBC; TSAT = (TI × 100)/TIBC.

ADVIA 2120 erythrocytes, reticulocytes and platelet indices, and other variables evaluated in the study are reported in the Additional file 1, including their abbreviations.

### Statistical analyses

Statistical analyses were performed using MedCalc statistical software (version 15.6; MedCalc Software, Ostend, Belgium). The D’Agostino-Pearson test was used to assess normal distribution of data. Data were reported as mean ± SD or median (minimum-maximum) based on their distribution. Comparisons between the two age groups were performed using the Mann-Whitney *U* test due to the non-Gaussian distribution of the majority of the data. Reference intervals were obtained using the 2.5th – 97.5th percentiles method following the Clinical and Laboratory Standards Institute (CLSI) guidelines for estimating percentiles and their 90% confidence intervals [21]. Outliers were identified with the Tukey test. Differences were considered to be statistically significant with *P* < 0.05.

## Results and discussion

For the haematological and chemical analyses, the number of samples available for each analyses, descriptive statistics, differences between groups and estimated RI in P5 and P30 are reported in Tables 1, 2 and 3. A significant increase in the circulating erythrocyte mass was detected in P30 compared to P5 as demonstrated by the higher values of Hb, HCT and RBC count. This finding was associated with a significant reduction in volume (MCV, MCVm) and a significant increase in anisocytosis (RDW, RDWm). Erythrocyte haemoglobin content indices (CH, CHm, MCH) were significantly lower in P30 compared to P5 with the exception of CHCM which was significantly higher in P30, while MCHC and CHCMm were not significantly different between groups (Table 1). Absolute reticulocyte count and percentage of reticulocytes were significantly lower in P30 compared to P5. Many other reticulocyte indices were significantly different between age groups (Table 2).

**Table 1:** Descriptive statistics, differences between groups and estimated reference intervals for haematological variables. Data are expressed as mean ± SD or median (minimum-maximum)

Variable	P5	P5	P5	P30	P30	P30	*P*-value
	Descriptive data	Reference Interval	*n*	Descriptive data	Reference Interval	*n*	
Hb (g/dL)	6.03 ± 1.02	3.56-7.74	61	8.84 ± 2.0	4.32-13.31	64	<0.0001
HCT (%)	20 ± 3	13-25	61	29 ± 6	16-41	64	<0.0001
CH (pg)	16.85 (14.10–22.30)	14.30–21.82	66	14.0 (9.0–19.0)	9.3–18.9	64	<0.0001
CHm (pg)	18.3 (16.1–22.4)	16.27–22.01	50	15.4 ± 1.5	12.7–18.8	33	<0.0001
CHDW (pg)	3.72 (2.90–5.84)	3.01–5.71	66	3.99 ± 0.66	2.66–5.33	64	0.3541
CHDWm (pg)	3.36 (2.82–5.47)	2.83–5.34	50	4.78 (2.61–5.55)	2.61–5.50	33	<0.0001
RBC (10^6^/μL)	3.26 ± 0.52	1.88–4.11	61	6.08 ± 0.93	4.08–8.17	64	<0.0001
MCV (fL)	61.97 ± 5.39	51.41–73.65	61	48.5 (32.4–61.5)	34.2–61.3	64	<0.0001
MCVm (fL)	65.9 ± 4.5	56.4–74.9	50	52.9 ± 3.9	45.3–60.5	33	<0.0001
MCHC (g/dL)	30.0. ± 1.6	26.1–32.7	61	29.9 ± 1.59	26.5–33.6	63	0.9606
MCH (pg)	18.50 ± 1.36	15.45–21.54	61	14.8 (9.1–20.2)	9.4–19.8	64	<0.0001
CHCM (g/dL)	27.35 ± 1.11	24.84–29.13	66	28.35 ± 1.60	25.13–31.62	64	0.0003
CHCMm (g/dL)	28.2 (26.7–30.8)	26.8–30.8	50	28.6 (26.8–30.9)	26.8–30.9	33	0.5389
RDW (%)	18.8 (15.8–25.7)	15.9–25.7	56	26.5 (13.5–38.5)	13.5–38.0	64	<0.0001
RDWm (%)	15.9 (14.2–25.7)	14.2–25.1	50	25.5 (12.7–32.2)	12.7–32.0	33	<0.0001
HDW (g/dL)	2.89 (2.28–4.13)	2.29–3.93	66	2.63 ± 0.40	2.02–3.50	64	<0.0001
HDWm (g/dL)	2.60 (2.14–3.68)	2.16–3.63	50	2.73 ± 1.99–3.28	1.99–3.28	33	0.1685
WBC (10^3^/μL)	7.47 (4.39–12.58)	4.50–12.55	58	11.6 ± 3.2	5.6–18.5	58	<0.0001
neutrophil (%)	44.71 ± 9.66	22.77–61.33	60	35.3 ± 15.0	10.8–70.6	63	0.0001
lymphocyte (%)	49.30 ± 9.49	33.45–70.86	60	57.9 ± 14.5	26.2–82.9	64	0.0005
monocyte (%)	3.11 ± 1.13	1.10–5.85	59	4.4 ± 1.5	1.4–8.3	64	<0.0001
eosinophil (%)	0.5 (0.1–3.1)	0–2.3	57	0.6 (0.1–7.6)	0–1.9	64	0.5699
basophil (%)	0.41 ± 0.16	0–0.79	60	0.4 (0.2–1.2)	0–0.9	63	0.1183
neutrophil (10^3^/μL)	3.17 ± 0.98	1.15–5.43	54	3.5 (0.8–9.9)	0.8–9.7	61	0.1472
lymphocyte (10^3^/μL)	3.85 ± 1.11	1.91–6.45	58	6.6 ± 2.4	2.7–12.8	60	<0.0001
monocyte (10^3^/μL)	0.23 (0.07–0.7)	0.075–0.68	59	0.4 (0.1–1.1)	0.1–1.1	59	<0.0001
eosinophil (10^3^/μL)	0.04 (0.01–0.48)	0–0.28	60	0.07 (0.01–0.38)	0.00–0.20	63	0.0014
basophil (10^3^/μL)	0.03 (0.01–0.2)	0–0.08	60	0.05 (0.02–0.33)	0.00–0.13	63	<0.0001
PLT (10^3^/μL)	594 (219–1142)	253–1286	61	503 ± 141	192–832	63	<0.0001
MPV (fL)	17.84 ± 3.78	9.99–25.32	61	8.5 (6.2–13.0)	6.5–12.7	63	<0.0001
PDW (%)	84.22 ± 8.32	69.17–100.30	61	60.40 (17.90–106.60)	18.59–101.98	64	<0.0001
PCT (%)	1.20 ± 0.50	0.29–2.52	61	0.40 (0.12–0.94)	0.18–0.91	62	<0.0001
MPC (g/dL)	24.5 (17.2–26.3)	17.81–26.30	61	24.09 ± 0.96	21.69–26.50	64	0.0182
MPM (pg)	2.84 ± 0.34	2.10–3.44	61	1.96 ± 0.22	1.58–2.45	63	<0.0001
PCDW (g/dL)	4.6 (4.0–5.7)	4.0–5.6	66	4.6 (3.8–5.9)	3.9–5.9	64	0.4354
PMDW (pg)	1.45 (0.99–1.62)	0.99–1.61	66	0.87 (0.61–1.55)	0.62–1.54	64	<0.0001

**Table 2:** Descriptive statistics, differences between groups and estimated reference intervals for reticulocyte indices. Data are expressed as mean ± SD or median (minimum-maximum)

Variable	P5	P5	P5	P30	P30	P30	*P*-value
	Descriptive data	Reference Interval	*n*	Descriptive data	Reference Interval	*n*	
Retic (10^9^cell/L)	369.3 ± 101.1	152.2–547.9	45	148.2 (53.4–554.5)	53.4–554.5	33	<0.000001
%Retic (%)	11.8 ± 3.8	4.6–20.7	49	2.5 (0.8–9.2)	0.8–9.2	33	<0.000001
MCVr (fL)	74.5 ± 7.3	60.7–89.4	48	56.5 ± 6.8	43.0–71.1	33	<0.000001
CHCMr (g/dL)	24.9 ± 0.9	23.4–27.0	48	26.1 ± 1.0	24.2–28.0	33	0.000008
CHr (pg)	18.1 (16.0–24.3)	16.2–23.8	48	14.6 ± 1.4	12.1–18.0	33	<0.000001
CHDWr (pg)	3.7 ± 0.3	3.1–4.4	48	3.5 (2.4–4.6)	2.4–4.6	33	0.166288
RDWr (%)	17.6 ± 2.1	14.2–20.7	48	19.5 (13.0–33.0)	13.0–33.0	33	0.055130
HDWr (g/dL)	3.36 ± 0.36	2.66–3.84	48	3.9 ± 0.62	2.7–5.1	33	0.000003
%Micro-r (%)	0.05 (0–0.8)	0.0–0.45	48	0.7 (0.0–15.5)	0.0–15.5	33	0.000005
%Macro-r (%)	26.5 (4.8–71.9)	6.7–70.7	48	2.6 (1.0–17.4)	1.0–17.4	33	<0.000001
%Hypo-r (%)	83.0 (62.8–94.3)	62.2–92.2	48	76.4 (57.2–85.8)	57.2–85.8	33	0.000036
%Hyper-r (%)	0.1 (0.0–0.3)	0.0–0.3	48	0.3 (0.0–2.7)	0.0–2.7	33	<0.000001
%LowCHr (%)	10.9 (0.5–35.4)	0.5–32.1	48	57.3 (6.0–76.8)	6.0–76.8	33	<0.000001
%HighCHr (%)	22.6 (9.2–76.3)	10.1–74.9	48	7.1 ± 2.5	3.3–13.0	33	<0.000001
CH delta (pg)	−0.1 ± 1.6	−3.3–2.1	48	−0.7 ± 0.7	−2.4–0.7	33	0.012741
CHCM delta (g/dL)	−3.4 ± 0.9	−5.2–(−1.8)	48	−2.6 ± 1.7	−5.3–0.8	33	0.075
CHDW delta (pg)	0.38 (−0.54–0.86)	−0.33–0.67	48	−1.0 ± 0.6	−2.19–(−0.05)	33	<0.000001
HDW delta (g/dL)	0.66 ± 0.27	0.12–1.07	48	1.26 ± 0.46	0.18–2.06	33	<0.000001
MCV delta (fL)	8.9 ± 6.6	−3.4–18.3	48	3.6 ± 4.4	−3.8–12.5	33	0.000255
RDW delta (%)	1.4 (−7.4–5.2)	−6.3–4.2	48	−3.8 ± 4.3	−12.6–3.0	33	<0.000001

**Table 3:** Descriptive statistics, differences between groups and estimated reference intervals for clinical chemistry. Data are expressed as mean ± SD or median (minimum-maximum)

Variable	P5	P5	P5	P30	P30	P30	*P*-value
	Descriptive data	Reference Interval	*n*	Descriptive data	Reference Interval	*n*	
Glucose (mg/dL)	124 (69–200)	71–196	49	111 ± 26	34–159	62	0.0016
Urea (mg/dL)	12.4 (4.9–31.7)	5.0–30.8	55	17 (4–40)	4–39	59	0.0002
Creatinine (mg/dL)	0.65 (0.30–0.88)	0.38–0.85	56	1.09 (0.31–1.41)	0.51–1.39	62	<0.0001
AST (U/L)	29 ± 7	10–47	56	31 (11–68)	13–65	62	0.9915
ALT (U/L)	23 ± 6	5–38	56	30 (11–58)	14–54	60	<0.0001
ALP (U/L)	3773 ± 1017	1324–6031	56	770 ± 270	110–1292	61	<0.0001
TP (g/dL)	5.0 ± 0.5	3.7–6.2	54	4.8 (1.2–6.7)	2.5–6.6	59	0.4464
Albumin (g/dL)	1.8 ± 0.3	1.0–2.6	55	3.0 (1.8–4.0)	1.9–4.0	59	<0.0001
A/G	0.56 (0.36–0.99)	0.37–0.98	53	1.5 ± 0.3	0.7–2.2	60	<0.0001
TI (μg/dL)	15 (9–31)	9–30	49	34 (7–157)	7–151	60	<0.0001
UIBC (μg/dL)	338 ± 72	218–524	44	326 (40–1058)	63–980	62	0.2031
TIBC (μg/dL)	354 ± 72	230–542	44	397 (128–882)	142–877	60	0.0031
TSAT (%)	4.2 ± 1.2	1.3–6.7	42	8.6 (0.8–55.6)	0.9–54	61	0.0058
Potassium (mEq/L)	4 ± 0.5	2.8–5.1	54	3.7 ± 0.4	2.9–4.6	63	0.0252
Sodium (mEq/L)	135 (126–140)	126–140	53	136 ± 4.7	125–147	63	0.2448

Circulating platelet number (PLT, PCT) was significantly decreased in P30 compared to P5; these results were associated with a significant reduction in platelet volume and mass (MPV, MPM) in P30 animals.

In the chemistry analysis, P5 subjects had significantly lower values of creatinine, urea, ALT, albumin and A/G and significantly higher ALP and potassium compared to P30 animals. Total iron concentration and TSAT % were significantly higher in P30 piglets compared to P5 (Table 3).

Swine are probably one of the most important models for translational medicine [7], and therefore a complete and accurate knowledge of their physiology should be mandatory. This kind of knowledge would represent a big improvement when it comes to refinement of animal experiments. When using commercial hybrids for scientific purposes, it is important to acknowledge that those animals are the products of strong zootechnical manipulation that constantly requires higher breeding and production performance [18]. Moreover, very young piglets (first month of age) present high variability in many clinical and clinicopathological variables due to rapid growth, nutrition and other metabolic conditions, some which are potentially related to iron status [15, 20]. As in other species, new-born and young piglets are extremely different than adult animals regarding laboratory results [20], thus, the determination of reliable age-related reference intervals for both haematology and chemistry variables in 5- and 30-day-old piglets is warranted. It is important to acknowledge the fact that our results may not perfectly translate to every other pigs’ breeds, but still represent a valid and robust general reference especially because obtained by one of the most common hybrid cross in Europe. Another important issue to address, before the discussion of the results, is the feeding protocol: P5 only received milk from the sows, while P30 were weaned at 28 days of life, therefore only ate solid feed for 2 days before blood sampling. This difference in alimentation may have contributed to some of the differences alongside with evolution of the gastro-intestinal system.

The results reported in this study showed high variability in the blood profiles among P5 and P30 animals. Unfortunately, it is hard to compare our data with the ones previously described in the literature mainly because of the poor or absent age distinction and the different types of animals used. However, our P5 and P30 animals had lower Hb, HCT, RBC, MCV and MCH values compared to animals of similar ages (days 6 and 36 from birth, respectively) [20].

In our study, P5 animals had a RBC regenerative response that was significantly reduced in P30, as further demonstrated by the results of reticulocyte indices (significantly higher Retic, %Retic, MCVr, %Macro-r and MCV delta). Similar findings have been reported previously and could represent a physiological response in the new-born pigs [15, 20]. However, a condition of iron deficiency could not be excluded in our piglets. It is well known that piglets can suffer from iron deficiency due to many causes such as immature iron metabolism, decreased iron intake or absorption and rapid growth. Iron deficiency can be associated with reduced weight gain, anaemia and even with increased mortality in these animals [22]. For these reasons, iron supplementation is highly recommended and the benefit of this treatment is well documented [18]. The role of iron deficiency in P5 animals, although suspected based on haematological results, could not be demonstrated with the current study design. In the initial phase, iron deficiency is characterised by enhanced erythropoiesis and even a regenerative anaemia, while microcytosis and hypochromasia (reduction of MCV and MCHC) are late findings associated with iron deficiency in animals and humans [23, 24]. The P30 animals that were supplemented with parenteral iron within the first 72 h after birth had a decreased reticulocyte response compared to the new-born animals. In addition, the results of iron profile parameters, particularly TI and TSAT, supported the potential role of iron deficiency in the piglets included in the present study. Total iron and TSAT values in P5 and even P30 animals were extremely decreased compared to the adult pig values reported in the literature [25]. However, TI and TSAT values were significantly higher at 30 days after birth, compared to new-born piglets. A previous study, using a different schedule of iron treatment, reported different results with particular regard to the iron profile parameters, compared to our study [15]. In our study, the upper limit for TI in P30 animals was five times higher than the P5 upper limit, however the lower limit was similar; the same happened for TSAT. The TIBC showed a slightly different trend, with a wider interval and both lower and higher values in P30 compared to P5. The overall pattern shown by iron-related parameters indicated that circulating iron was very low in these healthy animals. For this reason iron deficiency is hard to investigate in piglets and the determination of other iron-related variables such as ferritin, which is considered the main iron storage protein, may be helpful. Further studies on non-treated animals should be performed in order to investigate and quantify the physiological erythroid response of growing pigs. However, it is possible that an enhanced erythropoiesis accompanied by a relative iron deficiency may be considered a paraphysiological condition for this type of piglet in the first month after birth.

Swine are known to have an elevated platelet count compared to other animals, with frequent platelet clumping upon blood smear examination [20]. Although the total platelet count at both experimental times was comparable to adult animal values available in the literature, PLT, volume and mass were significantly reduced in P30 pigs compared to the new-born piglets. Similar data for young pigs are lacking in veterinary medicine to the best of our knowledge. Analogous to erythropoiesis, an enhanced thrombocytopoiesis that decreases shortly after birth could be explained by the increased need for platelets due to rapid growth or even iron deficiency. Iron deficiency, in fact, is recognised as a stimulus for the bone marrow to produce and release platelets, leading to thrombocytosis in small animals and humans [26].

The P5 and P30 total WBC count results in our study were consistent with the literature: neutrophil percentage decreased from P5 to P30 while the neutrophil total number did not differ significantly. On the contrary, the lymphocyte count and percentage were significantly increased in older animals, as previously reported [20].

Many chemistry variables evaluated were significantly different between the two age groups. These differences may impact the RI definition, however, it is difficult to clarify the exact physiological significance of these changes from an observational study. Interestingly, new-born swine may have a very low concentration of albumin that was significantly higher in 30-day-old animals, while TP concentration did not differ significantly between groups. As supported by the increase in A/G in P30 piglets, albumin concentration increased with the growth of these animals and this process was associated with a progressive reduction in the globulin fraction. Similar findings are lacking in previous studies.

Creatinine and urea values were significantly higher in P30 animals compared to P5, however both values were fully comparable to the adult values available in the veterinary literature [27]. In the authors’ opinion, this is the result of the fast growing rate of pigs leading to a rapid increase in muscle mass, helped by the gradual supplementation of enriched solid diet throughout the lactation period.

Another significant difference can be noticed in the ALP concentration, which was extremely higher in younger animals. The literature suggests that this finding is related to higher osteoblast activity in young, growing animals; nevertheless the exact role of ALP in swine in poorly understood, and its clinicopathological usefulness needs to be clarified in further studies [28, 29].

Age-related reference intervals for haematological and biochemical variables in wild boars have been recently published [30]. Although a similar automated haematology system (ADVIA 120) was used in that study, animals from zero to six months were referred to as piglets, and therefore a comparison of their data with our results would not be accurate or reliable. This different stratification of the population in their study design reflects changes in chemical variables as well, and their data seem to be more comparable to our P30 results.

## Conclusions

In conclusion, this paper highlights the high variability in haematological and chemistry variables between new-born and 30-day-old pigs, and between these animals and adult pigs. Moreover, our study provides specific age-related reference intervals for healthy commercial hybrid piglets that can be used as physiological standards for both translational and swine medicine. Age-related reference intervals will help in the correct interpretation of experimental results and should be considered an important step towards a more in-depth knowledge and standardisation of the swine animal model.

## Additional file

Additional file 1: Haematological and Biochemical variables evaluated in the study. (DOCX 18 kb)
