# Supplementary material for: The biomedical piglet: establishing reference intervals for haematology and clinical chemistry parameters of two age groups with and without iron supplementation
Source: BMC Vet Res. 2017 Jan 17;13:23. doi: 10.1186/s12917-017-0946-2 (PMC5240404; doi:10.1186/s12917-017-0946-2)
Supplement: Additional file 1: — Haematological and Biochemical variables evaluated in the study. (DOCX 18 kb) [file 12917_2017_946_MOESM1_ESM.docx]

Additional file 1. Haematological and Biochemical variables evaluated in the study.

| **Abbreviations** | **Description** |
| --- | --- |
| *Haematology* |  |
| RBC count | Red blood cells count |
| HCT | Haematocrit value |
| Hb | Haemoglobin concentration |
| CH | Cellular haemoglobin content |
| CHm | Cellular haemoglobin content of mature RBCs |
| CHDW | Cellular haemoglobin distribution width |
| CHDWm | Cellular haemoglobin distribution width of mature RBCs |
| MCV | Mean corpuscular volume |
| MCVm | Mean corpuscular volume of mature RBCs |
| MCHC | Mean corpuscular haemoglobin concentration |
| MCH | Mean corpuscular haemoglobin |
| CHCM | Corpuscular haemoglobin concentration mean |
| CHCMm | Corpuscular haemoglobin concentration mean of mature RBCs |
| HDW | Haemoglobin concentration distribution width |
| HDWm | Haemoglobin concentration distribution width of mature RBCs |
| RDW | RBC distribution width |
| RDWm | Mature RBC distribution width |
| WBC | Total white blood cell count |
| *Platelet indices* |  |
| PLT | Platelet count |
| MPV | Mean platelet volume |
| PDW | Platelet volume distribution width |
| PCT | Plateletcrit |
| MPC | Mean platelet component |
| PCDW | Platelet component distribution width |
| MPM | Mean platelet mass |
| PMDW | Platelet mass distribution width |
| *Reticulocytes indices* |  |
| Retic | Absolute reticulocyte count |
| %Retic | Percentage of reticulocytes |
| MCVr | Average size of reticulocytes |
| CHCMr | Average cell haemoglobin concentration of reticulocytes |
| CHDWr | Cellular haemoglobin distribution width of reticulocytes |
| CHr | Average haemoglobin content |
| RDWr | Distribution width of reticulocyte cell size |
| HDWr | Distribution width of CHCMr |
| %Micro-r | Percentage of microcytic reticulocytes |
| %Macro-r | Percentage of macrocytic reticulocytes |
| %Hypo-r | Percentage of hypochromic reticulocytes |
| %Hyper-r | Percentage of hyperchromic reticulocytes |
| %LowCHr | Percentage of reticulocytes with a low CH |
| %HighCHr | Percentage of reticulocytes with a high CH |
| CH delta | CHr-CHm |
| CHCM delta | CHCMr-CHCMm |
| CHDW delta | CHDWr-CHDWm |
| HDW delta | HDWr-HDWm |
| MCV delta | MCVr-MCVm |
| RDW delta | RDWr-RDWm |
| *Chemistry* |  |
| AST | Aspartate transaminase |
| ALT | Alanine transaminase |
| ALP | Alkaline phosphatase |
| TP | Total proteins |
| A/G | Albumin to globulin ratio |
| TI | Total iron |
| UIBC | Unsaturated iron binding capacity |
| TIBC | Total iron binding capacity |
| TSAT | TIBC saturation |
